# Spontaneous Rupture of a Hypopharyngeal Cyst in an Elderly Bedridden Patient

**DOI:** 10.7759/cureus.101409

**Published:** 2026-01-13

**Authors:** Assadiq E Ahmed, Alaa Q Abdalhafiz

**Affiliations:** 1 Otolaryngology — Head and Neck Surgery, Kalba Hospital, Kalba, ARE

**Keywords:** case report, dysphagia, elderly patient, hypopharyngeal cyst, laryngeal cyst, pyriform sinus cyst, spontaneous rupture

## Abstract

Hypopharyngeal cysts are rare benign lesions that may present with dysphagia and, less commonly, airway compromise and are typically managed surgically. We report the case of an 80-year-old bedridden man with multiple comorbidities who presented with progressive dysphagia due to a large hypopharyngeal cyst causing mechanical obstruction. Surgical intervention was recommended but declined by the patient’s family due to the patient’s frailty. Unexpectedly, the cyst spontaneously ruptured during hospitalization, resulting in immediate and complete resolution of dysphagia without aspiration, respiratory compromise, or other complications. Follow-up endoscopy and imaging confirmed resolution of the lesion and normalization of hypopharyngeal anatomy. This case represents a rare and unexpected clinical scenario and contributes to the limited literature on the natural history of hypopharyngeal cysts. It highlights that, in carefully selected high-risk patients with a stable airway, conservative observation may be a reasonable management option when surgical intervention is contraindicated.

## Introduction

Hypopharyngeal cysts are rare benign lesions that arise from the mucosal lining or minor salivary glands of the hypopharynx [1,2]. They are typically asymptomatic but may become clinically significant when they enlarge and compress adjacent structures, leading to symptoms such as dysphagia, odynophagia, voice changes, or airway compromise [3,4]. Despite their benign nature, hypopharyngeal cysts are clinically important due to their potential to cause progressive dysphagia and, in rare cases, airway obstruction, particularly in elderly or medically fragile patients. Diagnosis is often established either intentionally or incidentally through endoscopic evaluation or imaging studies. Surgical excision remains the standard treatment [5]; however, limited data exist regarding the natural history and non-surgical outcomes of these lesions in patients who are poor surgical candidates. Management must therefore be individualized, especially in elderly or high-risk populations where comorbidities may preclude operative intervention. This case report describes an unusual but clinically significant instance of spontaneous rupture of a hypopharyngeal cyst in an elderly, nonoperative patient with immediate symptom resolution.

## Case presentation

An 80-year-old man had a complex medical history, including hypertension, dyslipidemia, and a prior diagnosis of deep venous thrombosis treated in 2013. He was also a known case of benign prostatic hyperplasia managed with an indwelling urinary catheter, chronic obstructive pulmonary disease on home nebulizer therapy, and cervical and lumbar disc disease status post spinal surgery. As a result, the patient had been bedridden for a prolonged period.

He was brought to the emergency department after a choking episode while eating. At home, he was found to be drowsy with oxygen desaturation at 85% and was transported by the National Ambulance. On arrival to the emergency department, the patient was hemodynamically stable and maintaining oxygen saturation above 95% on room air, with blood pressure within normal limits. The initial episode of drowsiness and desaturation occurred during the choking event at home and had resolved prior to hospital assessment. He reported a history of progressive dysphagia over several weeks, predominantly affecting solid food intake, with reliance on liquids and semisolids.

At presentation, the patient was not in acute respiratory distress and had no stridor, voice change, or external neck swelling. Airway assessment suggested a stable but significant mechanical obstruction rather than an evolving acute airway crisis.

**Figure 1:**
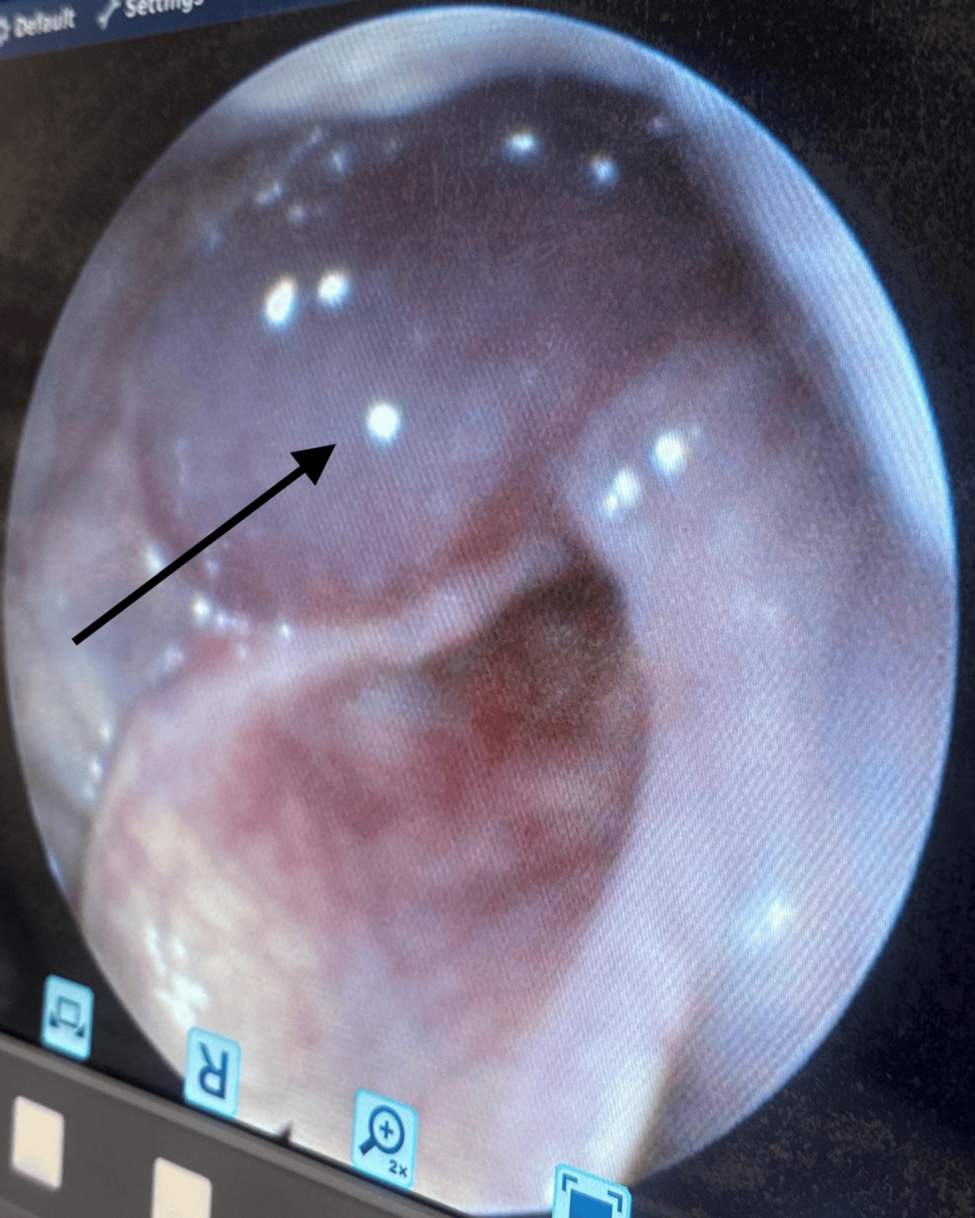
Pre-rupture endoscopic view showing a smooth, cystic swelling arising from the right hypopharynx (black arrow), pushing the supraglottic structures medially

**Figure 2:**
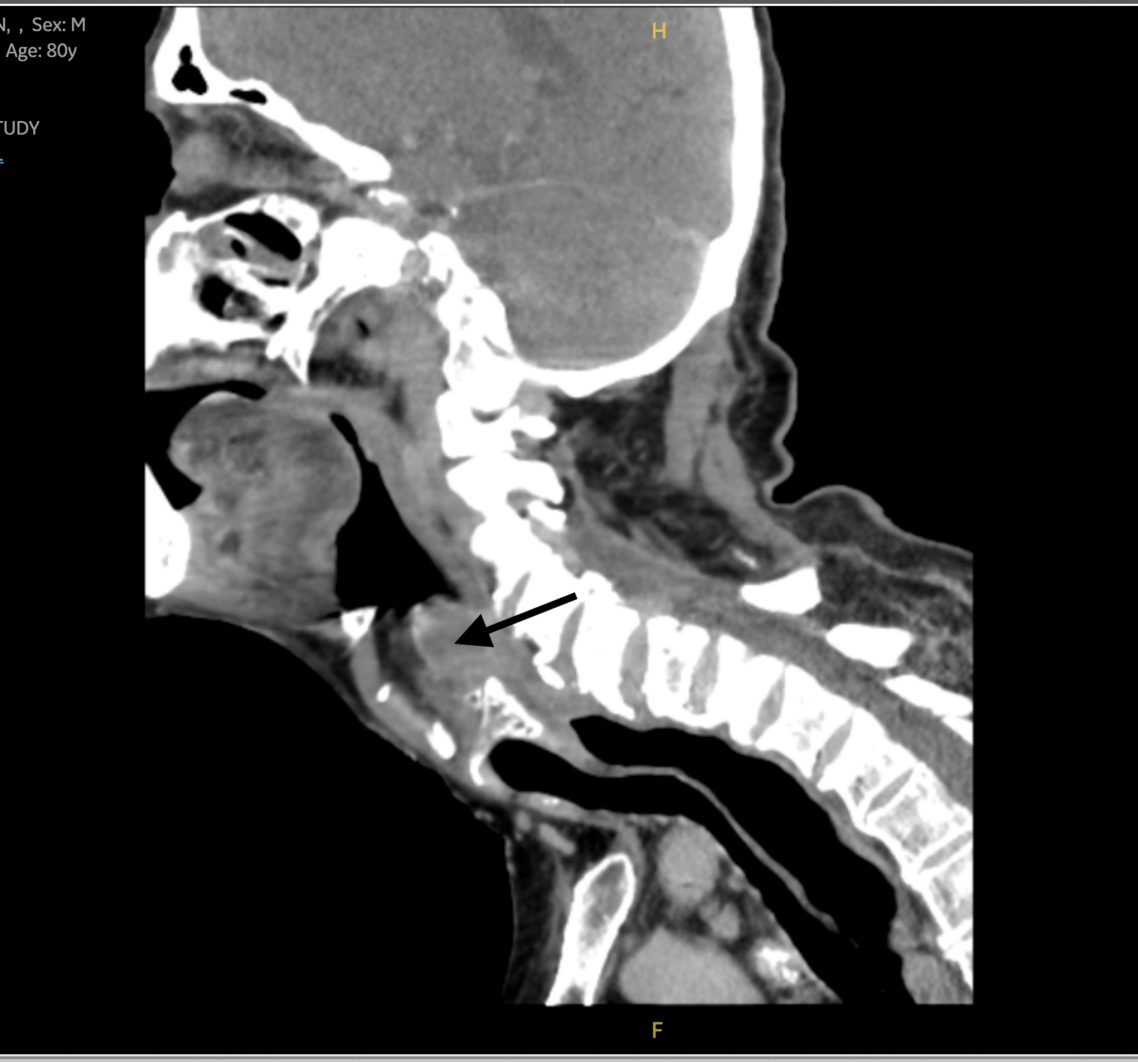
Sagittal view of the CT scan showing a hypopharyngeal cyst (black arrow) compressing the upper aerodigestive tract

**Figure 3:**
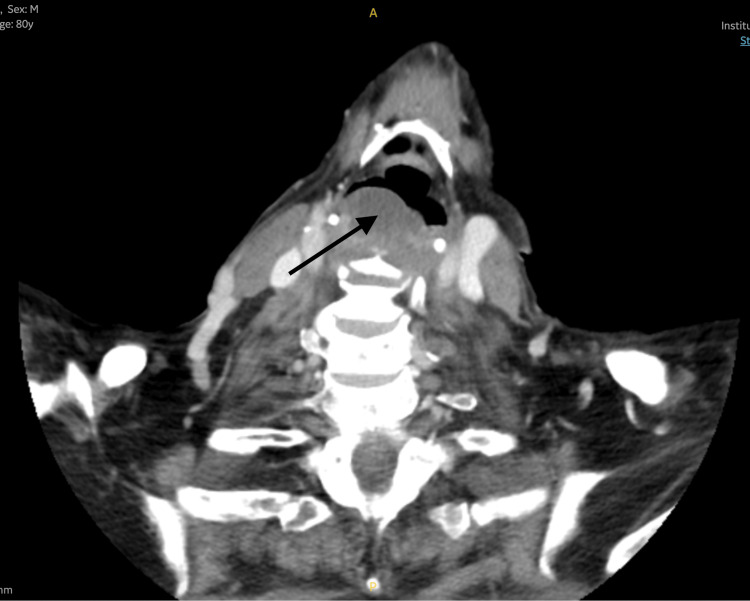
Axial view of the CT of the neck confirming a cystic lesion (black arrow) on the right side of the hypopharynx displacing the airway medially

Flexible laryngoscopy revealed a large right-sided hypopharyngeal cystic lesion displacing the supraglottic structures medially (Figure 1). A CT scan confirmed a well-defined hypopharyngeal cyst measuring 2.1 × 2.5 × 1.5 cm in anteroposterior, transverse, and craniocaudal dimensions, respectively, with significant narrowing of the upper aerodigestive tract (Figures 2-3). Despite the obstruction, the patient remained clinically stable and maintained adequate oxygenation on room air. Attempts at nasogastric tube insertion were unsuccessful due to mechanical obstruction by the cyst. Surgical excision was recommended, but declined by the patient and family.

**Figure 4:**
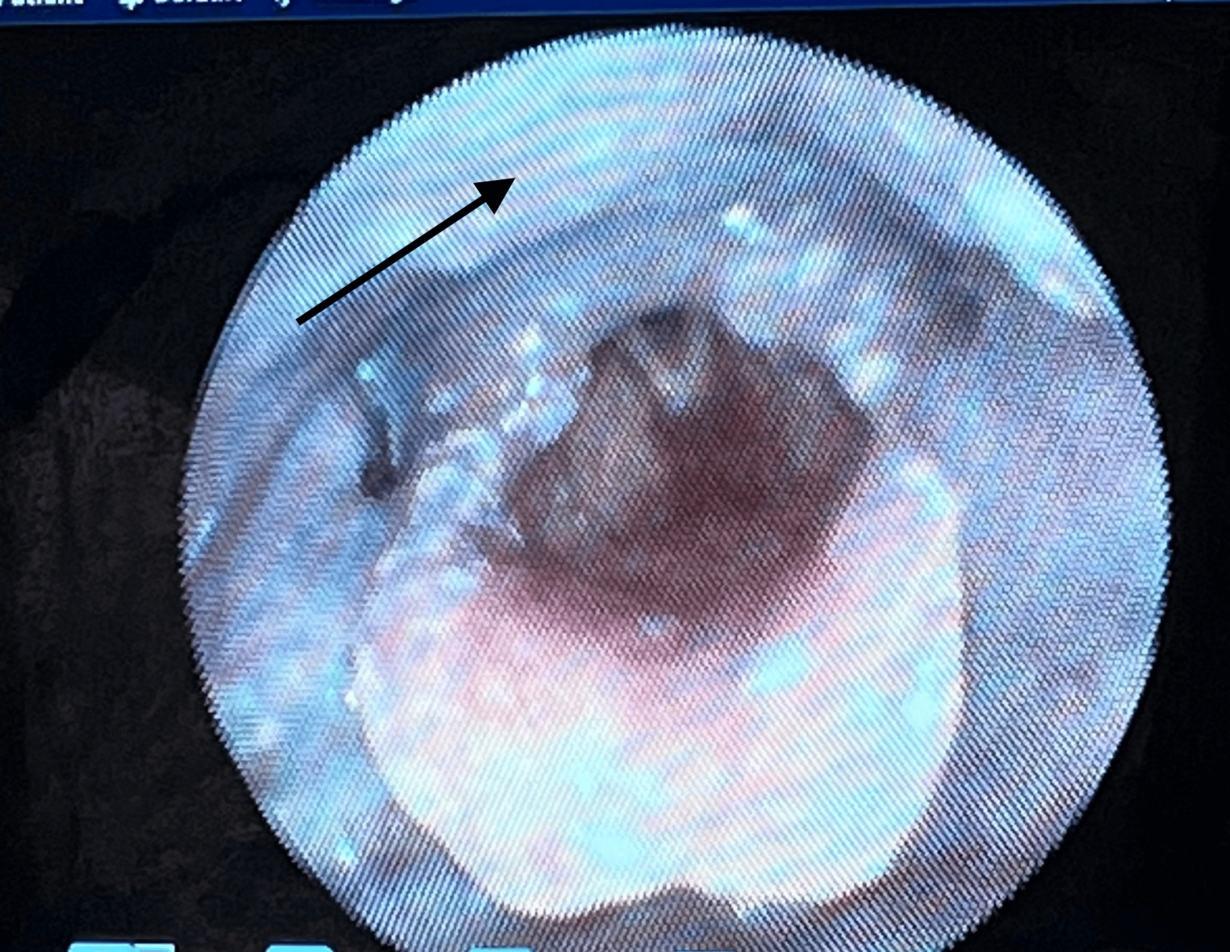
Post-rupture endoscopic view showing resolution of the cystic swelling (black arrow) with improved visualization of the supraglottic and hypopharyngeal anatomy

**Figure 5:**
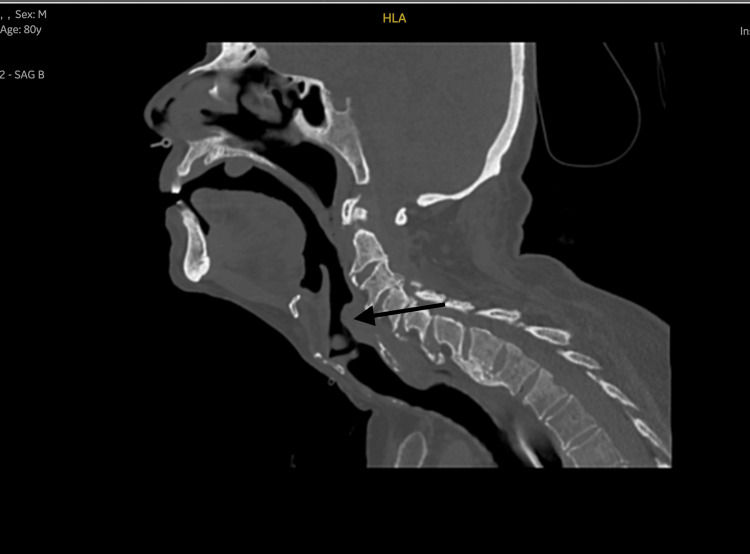
Post-rupture CT scan of the neck, sagittal view, showing resolution of the hypopharyngeal cyst (black arrow) and re-expansion of the airway

**Figure 6:**
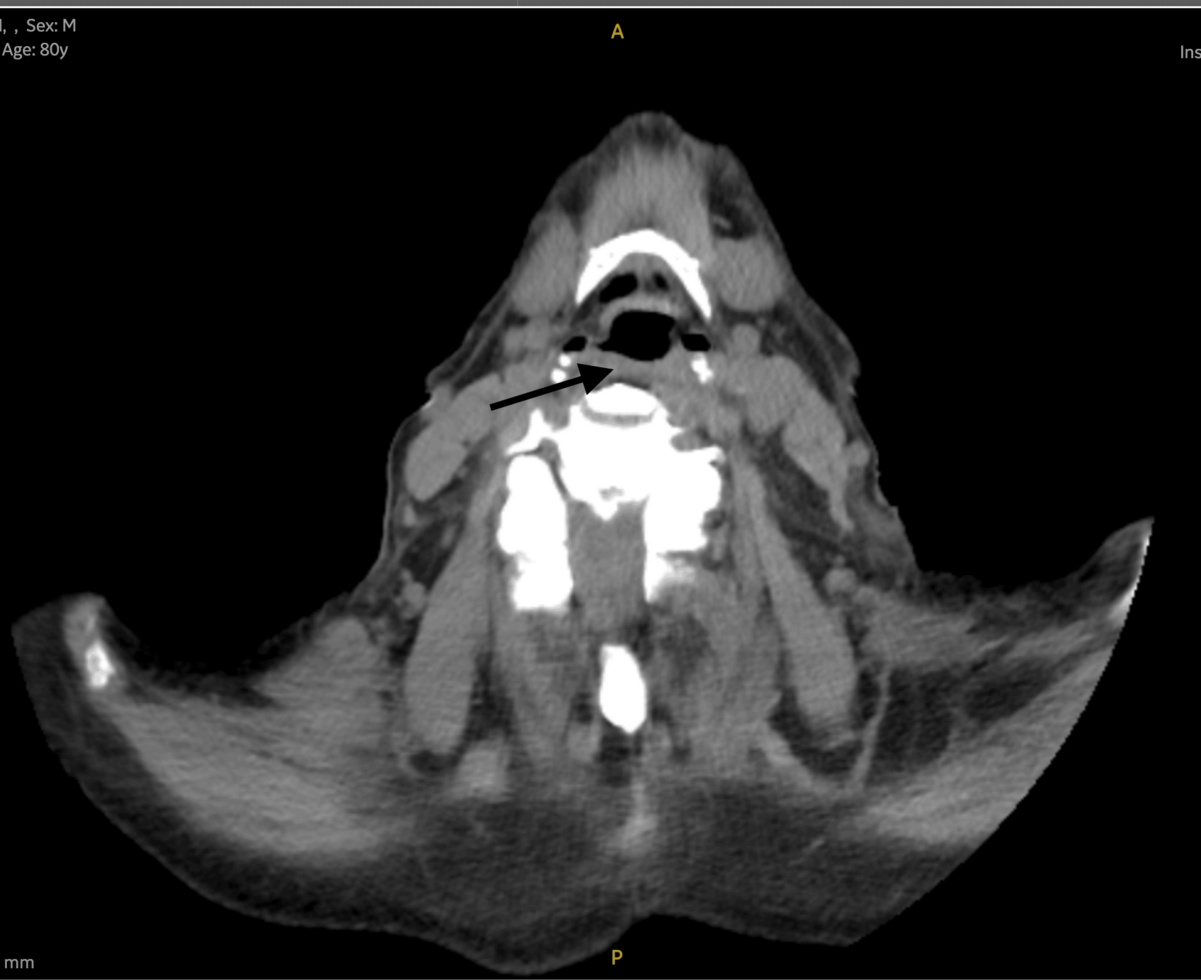
Post-rupture CT scan of the neck, axial view, confirming absence of the cyst (black arrow) and normalization of the hypopharyngeal contour

On the fifth day of admission, the patient unexpectedly reported marked improvement in swallowing. Repeat flexible endoscopy demonstrated collapse of the cystic cavity with no visible residual sac, findings consistent with spontaneous rupture (Figure 4). The patient did not report any abnormal taste sensation, coughing, choking, noisy breathing, or respiratory distress following the rupture and experienced immediate symptomatic relief. Follow-up CT imaging showed normalization of the hypopharyngeal anatomy (Figures 5-6). The patient resumed oral feeding and was discharged in stable condition after 72 hours of observation, with a scheduled follow-up appointment.

## Discussion

Hypopharyngeal cysts, though uncommon, are part of the differential diagnosis in patients presenting with dysphagia and upper aerodigestive symptoms. Pyriform sinus cysts account for approximately 5% of hypopharyngeal cysts [1]. It has been reported that 51% of laryngopharyngeal cysts originate from the epiglottis, with most occurring at the lingual surface [6]. Pyriform sinus cysts can occur at any age, although a higher prevalence has been observed in individuals aged 50 to 60 years [1]. These cysts include mucus retention cysts, ductal cysts, lymphoepithelial cysts, and congenital anomalies such as branchial cleft cysts. Symptomatic presentation typically correlates with the size and anatomical location of the lesion [1-3]. Flexible laryngoscopy and cross-sectional imaging (CT or MRI) are essential for diagnosis and surgical planning [4,7]. Imaging plays a key role in differentiating hypopharyngeal cysts from other lesions, such as tumors or abscesses, thereby ensuring accurate diagnosis [4].

Most previously reported hypopharyngeal cysts have been managed surgically or discovered incidentally. Conservative management is generally not favored due to the potential risks of airway obstruction, aspiration, or persistent dysphagia. Treatment is largely size-dependent [5]. Small, asymptomatic pharyngeal retention cysts typically do not require resection [2], whereas larger cysts or those causing symptoms usually warrant intervention. Transoral endoscopic excision remains the standard treatment and is generally curative with low recurrence rates [5]. Marsupialization may be sufficient in selected cases, and decompression by puncture and drainage can facilitate complete excision of larger cysts [2,8]. Xu et al. reported that intraoperative aspiration of cyst contents reduces internal pressure and facilitates identification of the cyst base during surgical management [9]. More recently, transoral robotic surgery has been described as an alternative approach, offering enhanced visualization and instrument maneuverability for safe and complete excision of hypopharyngeal lesions [10].

Cyst rupture most commonly occurs during surgical manipulation [1]. Incidental rupture, particularly during airway instrumentation or intubation, may be associated with serious complications, including aspiration and airway compromise [11]. Aspiration of vallecular cysts prior to intubation has been described as a strategy to reduce cyst size and facilitate airway management [8,12].

In elderly patients with significant comorbidities or in those who decline surgery, alternative management strategies must be considered. In the present case, surgical intervention was declined due to the patient’s frail condition, and the cyst subsequently ruptured spontaneously without post-rupture complications. As no surgical intervention or biopsy was performed, histopathological classification of the cyst remains presumptive and is based on characteristic endoscopic and radiological findings. To our knowledge, this is the first reported case in which spontaneous rupture of a hypopharyngeal cyst resulted in immediate and complete symptom resolution without intervention, offering novel insight into the potential natural course of these lesions. This observation suggests that conservative observation may be considered in carefully selected high-risk patients, although further reports are needed to support this approach. The case also highlights the importance of individualized decision-making and shared discussions when balancing procedural risks against quality-of-life considerations in elderly patients.

The mechanism underlying spontaneous cyst rupture is not fully understood but is thought to involve increased internal pressure, potentially exacerbated by swallowing or minor trauma. Future studies may help identify predictive factors or imaging features associated with spontaneous resolution. In this patient, concordant endoscopic and radiological evidence of a mechanically obstructing hypopharyngeal cyst, followed by documented post-rupture anatomical resolution and symptom improvement, provides direct support for the conclusion that conservative observation may be a reasonable interim strategy in carefully selected high-risk patients with a stable airway.

## Conclusions

Hypopharyngeal cysts, although rare, should be considered in elderly patients presenting with progressive dysphagia. While surgical excision remains the standard of care, spontaneous rupture can occur and may result in full symptom resolution. Early assessment with flexible laryngoscopy and cross-sectional imaging is recommended to accurately define the lesion, assess the degree of airway compromise, and guide management planning. In patients with significant comorbidities who are poor surgical candidates or decline operative intervention, careful non-surgical observation may be considered, provided that the airway remains stable and close clinical monitoring is ensured. In frail or unfit patients, conservative observation under close clinical monitoring may be an acceptable interim strategy. Clinicians should remain vigilant for changes in symptoms that may indicate spontaneous cyst rupture or potential complications, and appropriate documentation and follow-up are essential to detect recurrence, infection, or delayed adverse events. This case contributes to the limited literature on non-surgical outcomes in hypopharyngeal cysts and emphasizes the importance of patient-centered care.
